# Sleep, Glial Function, and the Endocannabinoid System: Implications for Neuroinflammation and Sleep Disorders

**DOI:** 10.3390/ijms25063160

**Published:** 2024-03-09

**Authors:** Josué Camberos-Barraza, Alejandro Camacho-Zamora, José C. Bátiz-Beltrán, Juan F. Osuna-Ramos, Ángel R. Rábago-Monzón, Marco A. Valdez-Flores, Carla E. Angulo-Rojo, Alma M. Guadrón-Llanos, Verónica J. Picos-Cárdenas, Loranda Calderón-Zamora, Claudia D. Norzagaray-Valenzuela, Feliznando I. Cárdenas-Torres, Alberto K. De la Herrán-Arita

**Affiliations:** 1 Faculty of Medicine, Autonomous University of Sinaloa, Culiacán 80019, Mexico; 2 Faculty of Biology, Autonomous University of Sinaloa, Culiacán 80019, Mexico; 3 Faculty of Nutrition Sciences and Gastronomy, Autonomous University of Sinaloa, Culiacán 80019, Mexico

**Keywords:** brain health, endocannabinoid system, glia, inflammation, sleep disorders

## Abstract

The relationship between sleep, glial cells, and the endocannabinoid system represents a multifaceted regulatory network with profound implications for neuroinflammation and cognitive function. The molecular underpinnings of sleep modulation by the endocannabinoid system and its influence on glial cell activity are discussed, shedding light on the reciprocal relationships that govern these processes. Emphasis is placed on understanding the role of glial cells in mediating neuroinflammatory responses and their modulation by sleep patterns. Additionally, this review examines how the endocannabinoid system interfaces with glia-immune signaling to regulate inflammatory cascades within the central nervous system. Notably, the cognitive consequences of disrupted sleep, neuroinflammation, and glial dysfunction are addressed, encompassing implications for neurodegenerative disorders, mood disturbances, and cognitive decline. Insights into the bidirectional modulation of cognitive function by the endocannabinoid system in the context of sleep and glial activity are explored, providing a comprehensive perspective on the potential mechanisms underlying cognitive impairments associated with sleep disturbances. Furthermore, this review examines potential therapeutic avenues targeting the endocannabinoid system to mitigate neuroinflammation, restore glial homeostasis, and normalize sleep patterns. The identification of novel therapeutic targets within this intricate regulatory network holds promise for addressing conditions characterized by disrupted sleep, neuroinflammation, and cognitive dysfunction. This work aims to examine the complexities of neural regulation and identify potential avenues for therapeutic intervention.

## 1. Introduction

Sleep, a fundamental and enigmatic physiological process, is integral to maintaining optimal cognitive function and overall well-being. Recent research has unveiled a complex interplay between sleep regulation, glial function, the immune system, and the endocannabinoid system (ECS), with profound implications for neural homeostasis [1,2,3,4,5].

The ECS, comprising endogenous cannabinoids, receptors, and enzymes, has emerged as a critical modulator of sleep patterns, influencing both the initiation and maintenance of sleep [4,6]. Concurrently and once considered tolerant support cells, glial cells are now recognized as active contributors to neuroinflammatory processes and synaptic regulation [7]. The intricate relationships between the ECS and glia-mediated immune responses in the context of sleep regulation present a novel avenue for understanding the broader implications of disrupted sleep on neural health.

Our understanding of the communication between sleep and the immune system has expanded, highlighting the role of glial cells as mediators of neuroinflammatory responses during sleep disturbances. Elucidating the molecular mechanisms through which sleep patterns influence glial function and, reciprocally, how glial cells modulate sleep-related processes holds the key to comprehending the broader consequences for cognitive function.

In this context, exploring the cognitive implications of altered sleep, neuroinflammation, and glial dysfunction becomes paramount. The intricate relationships within this triad significantly impact cognitive processes and may underlie the pathophysiology of neurodegenerative disorders, mood disturbances, and cognitive decline. Thus, unraveling the mechanisms governing cognitive consequences becomes crucial for both basic neuroscience and clinical applications.

As we delve into this complex interaction, we aim to provide a comprehensive overview of the relationships among sleep, glial function, the immune system, and the ECS. By elucidating the molecular intricacies and exploring the cognitive implications, this review aims to contribute to our evolving understanding of neural regulation and identifies potential avenues for therapeutic interventions aimed at restoring homeostasis in conditions marked by disrupted sleep and neuroinflammation.

## 2. The Neurobiology of Sleep

The sleep–wake cycle (SWC) is a complex and intricate process governed by the concerted activity of various nuclei and cells within the central nervous system (CNS). The circadian regulation of SWC manifests as an intricate molecular process, where the suprachiasmatic nucleus (SCN) in the hypothalamus assumes the role of a master conductor, coordinating molecular events to synchronize the organism’s internal clock with the external photoperiod [1,8,9].

### 2.1. Circadian Sleep Regulation

The stimulation of retinal cells by photons to activate the SCN involves a complex process known as phototransduction. The retinal cells responsible for this light detection are primarily the photoreceptor cells, namely rods and cones, located in the outermost layer of the retina. However, a subset of retinal ganglion cells contains a photopigment called melanopsin, which is particularly sensitive to blue light (400–495 nm). The process begins with the absorption of photons by the photopigments within these cells [10,11,12].

The activation and depolarization of melanopsin-expressing retinal ganglion cells (mRGCs) represent a distinct pathway in the phototransduction process that is independent of traditional rod and cone photoreceptors. The process begins with the absorption of photons by melanopsin in mRGCs. Melanopsin undergoes a conformational change upon photon absorption, leading to the activation of intracellular signaling cascades. Unlike the phototransduction in rods and cones, which involves a G protein-coupled pathway, melanopsin employs a more direct signaling mechanism [11,12,13].

Upon activation, melanopsin activates a class of ion channels known as transient receptor potential (TRP) channels, specifically TRPC6 and TRPM1. These channels allow the influx of cations, particularly sodium and calcium ions, into the mRGCs, leading to membrane depolarization. The depolarization of mRGCs results in the generation of action potentials. Unlike conventional retinal ganglion cells, which primarily transmit visual information related to images, mRGCs are specialized in conveying ambient light levels to brain regions involved in the regulation of circadian rhythms and other non-image-forming visual functions. These action potentials generated by depolarized mRGCs are transmitted along the retinohypothalamic tract, a direct neural pathway that projects to the SCN of the hypothalamus [14,15].

The SCN then integrates this photic input to synchronize the body’s circadian rhythm with the external light–dark cycle. The activation of glutamate receptors in the SCN initiates a cascade of intracellular events, including the induction of immediate early genes, such as c-Fos. Immediate early genes, in turn, contribute to the activation of signaling pathways that influence the Circadian Locomotor Output Cycles Kaput (CLOCK) and Brain and Muscle ARNT-like 1 (BMAL1) gene expression. The expression of CLOCK and BMAL1 genes is a fundamental aspect of the molecular machinery that governs the circadian rhythm, the internal biological clock that regulates various physiological and behavioral processes over a 24-hour cycle. These transcription factors heterodimerize and form a complex, which binds to specific DNA sequences known as E-box elements in the promoter regions of target genes, including their own promoters. Upon binding to the E-box elements, the CLOCK–BMAL1 complex promotes the transcription of target genes, including CLOCK and BMAL1 genes themselves. This leads to the synthesis of mRNA molecules for CLOCK and BMAL1. The transcriptional activation of these genes is a key initiating step in the circadian cycle. The newly synthesized CLOCK and BMAL1 mRNA molecules are then transported to the cytoplasm, where they undergo translation to produce the corresponding CLOCK and BMAL1 proteins. The translated proteins form a heterodimeric complex in the cytoplasm, preparing to re-enter the nucleus [16,17].

Once formed, the CLOCK–BMAL1 complex translocates back into the nucleus, where it binds to E-box elements in the promoter regions of its own genes, creating a self-regulating feedback loop. However, the circadian regulation does not stop there. The CLOCK–BMAL1 complex also induces the expression of genes encoding negative regulators, such as period (PER) and cryptochrome (CRY) genes. As the PER and CRY proteins accumulate in the nucleus, they eventually form a complex that inhibits the transcriptional activity of CLOCK–BMAL1. This negative feedback loop results in the suppression of CLOCK and BMAL1 gene expression, creating an oscillatory pattern with a roughly 24-hour period. The degradation of PER and CRY proteins over time releases the inhibition on CLOCK–BMAL1, allowing the transcriptional activation of CLOCK and BMAL1 genes to begin anew. This cyclic process repeats, generating the characteristic oscillations of CLOCK and BMAL1 gene expression that underlie the circadian rhythm [16,17,18,19].

The overall effect of external cues, particularly light, is to entrain the internal circadian rhythm to the external environment. This entrainment adjusts the phase and period of the circadian cycle to match the environmental day–night cycle, optimizing the alignment of internal biological processes with the demands of the external world.

### 2.2. Neural Dynamics of Sleep–Wake Transitions

Upon activation, the SCN neurons release glutamate, which acts on receptors in the lateral hypothalamus. When glutamate from the SCN binds to its receptors on neurons in the lateral hypothalamus, it triggers a series of events that lead to the release of hypocretin (also known as “orexin”). Hypocretin is a neuropeptide that plays a crucial role in promoting wakefulness and maintaining arousal. Hypocretin neurons have projections throughout the brain, and their activation promotes wakefulness and inhibits sleep-promoting pathways. In the context of the SWC, hypocretin is particularly important in maintaining wakefulness and preventing transitions to sleep. Its actions are primarily mediated through interaction with two G protein-coupled receptors, HCRTR1 and HCRTR2, expressed in various brain regions implicated in sleep–wake regulation. Upon binding to these receptors, hypocretin initiates a cascade of intracellular events. The activation of G proteins linked to HCRTR1 and HCRTR2 stimulates phospholipase C (PLC), leading to the hydrolysis of phosphatidylinositol 4,5-bisphosphate (PIP2) into inositol triphosphate (IP3) and diacylglycerol (DAG). This results in an increase in intracellular calcium levels, a crucial signaling event contributing to the excitatory effects of hypocretin. Simultaneously, hypocretinergic neurons modulate ion channels in target neurons. Hypocretin enhances the activity of excitatory ion channels, including sodium channels, promoting membrane depolarization and rendering the neuron more prone to generating action potentials. At the same time, inhibitory ion channels, such as potassium channels, are inhibited, further amplifying the excitatory effects of hypocretin [20,21,22,23].

The arousal-promoting effects of hypocretin extend to its influence on neurotransmitter release. Hypocretinergic neurons project widely throughout the brain, with notable connections to key arousal centers. Activation of hypocretin receptors leads to the release of neurotransmitters like norepinephrine, dopamine, serotonin, and acetylcholine. These neurotransmitters contribute to the overall enhancement of neuronal activity, reinforcing wakefulness. One significant target of hypocretin projections is the locus coeruleus, a brainstem nucleus releasing norepinephrine. The activation of this system by hypocretin further promotes arousal and vigilance. Additionally, hypocretinergic projections to the tuberomammillary nucleus stimulate the release of histamine, another neurotransmitter linked to wakefulness. Hypocretin’s influence on the cholinergic (pedunculopontine nuclei: PPT; laterodorsal tegmental nuclei: LDT) and glutamatergic systems further contributes to its wake-promoting effects. Through intricate interactions with these neurotransmitter systems, hypocretin fine-tunes the excitability of target neurons, facilitating a state of heightened arousal [24,25].

The transition between wakefulness and sleep is not a linear process but a dynamic interaction and integration of these neurotransmitter systems. Reciprocal inhibitory and excitatory connections weave a seamless tapestry, guiding the brain through the distinct stages of sleep. Sleep is a dynamic process characterized by distinct stages that follow a cyclical pattern throughout the night. The two main categories of sleep are non-REM (NREM) sleep and REM (rapid eye movement) sleep, each associated with unique physiological and neural characteristics [25].

As an individual transitions from wakefulness to sleep, the initial phase is NREM sleep, comprising stages N1, N2, and N3. Stage N1, also known as the transition to sleep or light sleep, typically lasts for a few minutes. The transition from wakefulness to N1 is marked by a reduction in muscle tone and a slowing of eye movements. Stage N2 follows N1 and is a more stable stage of light sleep. It constitutes a significant portion of total sleep time. In N2, theta waves dominate the electroencephalogram (EEG), and sleep spindles (short bursts of high-frequency activity) and K-complexes (sharp, high-amplitude waveforms) characterize the neural activity. These features are believed to play a role in sensory gating and memory consolidation. The deepest stage of NREM sleep is Stage N3, also referred to as slow-wave sleep (SWS) or deep sleep. This stage is characterized by the presence of slow-wave delta (delta) waves on the EEG. SWS is crucial for physical restoration and repair, as growth hormone is released during this stage, and various physiological processes, such as immune system functioning, are enhanced. After NREM sleep, the sleep cycle progresses into REM sleep. REM sleep is associated with vivid dreaming, rapid eye movements, and a profound suppression of voluntary muscle activity, leading to temporary paralysis, known as REM atonia. The neural activity during REM sleep resembles that of wakefulness, with rapid and desynchronized EEG patterns. Throughout the night, sleep cycles repeat approximately every 90 to 110 min, with NREM and REM stages occurring in succession. The proportion of time spent in each sleep stage varies across the night, with a higher proportion of SWS and REM sleep in the first half of the night and more NREM Stage 2 in the later cycles [26,27,28,29].

This cyclical pattern of sleep stages is orchestrated by the interaction of neural circuits and neurotransmitters within the brain, particularly involving the thalamus, hypothalamus, and brainstem nuclei. The orchestrated transitions between NREM and REM sleep contribute to the overall restorative and regulatory functions of sleep, influencing cognitive, emotional, and physiological well-being.

### 2.3. Mechanisms Governing the Transition from Wakefulness to Sleep

As wakefulness transitions to sleep, several key molecular mechanisms orchestrate the onset of NREM sleep. 

The release of melatonin, a hormone primarily synthesized by the pineal gland, is intricately regulated by the circadian system and influenced by external light cues. The synthesis and release of melatonin are part of the body’s circadian rhythm, serving as a key player in the regulation of the SWC. When exposed to darkness or low-light conditions, mRGCs signal the SCN. The SCN, in turn, relays signals to the pineal gland, signaling the onset of darkness and the impending night. The pineal gland responds to these signals by increasing the production of melatonin from its precursor, serotonin. Melatonin is released into the bloodstream and peaks during the night, reaching its highest levels in the middle of the night. This surge in melatonin contributes to the physiological changes associated with the promotion of sleep, such as a decrease in body temperature and an increase in feelings of sleepiness [30].

Melatonin exerts its effects by binding to specific receptors, known as melatonin receptors, which are widely distributed in various tissues throughout the body. The two primary subtypes of melatonin receptors are MT1 and MT2. When melatonin receptors are activated, particularly in the brain and the SCN, they initiate a cascade of intracellular events. The activation of these receptors inhibits the production of cyclic AMP (cAMP), a second messenger involved in signal transduction. The reduction in cAMP levels leads to the modulation of various cellular processes, ultimately contributing to the inhibitory effects of melatonin on neural activity and promoting the physiological changes associated with sleep [31,32]. In addition to its role in the circadian regulation of sleep, melatonin also has antioxidant and immunomodulatory properties [33,34]. The activation of melatonin receptors in various tissues influences cellular functions beyond the regulation of the sleep–wake cycle. 

Another crucial aspect for NREM sleep is the intrinsic rhythmic activity of VLPO neurons. As an individual transitions from wakefulness to sleep, these neurons exhibit increased firing rates. The intrinsic rhythmicity of VLPO neurons is influenced by various factors, including neuromodulators, and contributes to the release of GABA. In addition, neuromodulators such as adenosine play a significant role in the regulation of GABA release in the VLPO. Adenosine levels in the brain accumulate during wakefulness and decline during sleep. Adenosine binds to receptors on VLPO neurons, enhancing GABAergic transmission. The inhibitory actions of GABA released from the VLPO contribute to the dampening of arousal-promoting circuits, facilitating the transition to sleep [35,36,37].

An additional key component in the transition to NREM sleep is the thalamus. During wakefulness, the thalamus relays sensory information to the cortex, contributing to alertness. As sleep onset approaches, thalamic relay neurons undergo a hyperpolarization, decreasing their responsiveness to sensory input. This thalamic “gate” closure limits the flow of external stimuli to the cortex, facilitating the transition to a more internally driven state characteristic of NREM sleep. Simultaneously, the cortex undergoes changes in neural activity. Slow-wave activity, characterized by synchronized, high-amplitude, low-frequency oscillations, becomes prominent. This slow-wave activity is associated with the deepest stages of NREM sleep and is thought to be crucial for restorative processes, including memory consolidation and synaptic homeostasis [38,39,40].

The transition from NREM to REM sleep is a complex process regulated by specific nuclei, neurotransmitters, and neural pathways within the brainstem and forebrain. This intricate orchestration involves a delicate balance between inhibitory and excitatory neurotransmitter systems. The pontine tegmentum, specifically the sublaterodorsal nucleus (SLD) and the ventral periaqueductal gray (vPAG), is crucial for the initiation and maintenance of REM sleep. These nuclei serve as the primary generators of REM sleep-related neural activity [12,26]. This transition begins with the decline of activity in NREM-related nuclei. As the arousal systems, such as the locus coeruleus and the dorsal raphe nucleus, become less active, the inhibitory influence on REM-promoting regions is alleviated. The locus coeruleus, which releases norepinephrine during wakefulness and NREM sleep, decreases its firing rate as sleep progresses. Meanwhile, cholinergic neurons in the PPT and the LDT become increasingly active. The cholinergic output from the PPT and LDT promotes the desynchronization of neural activity in the forebrain, leading to the characteristic features of REM sleep, such as rapid eye movements and vivid dreaming. Additionally, serotonergic neurons in the dorsal raphe nucleus decrease their firing rate during REM sleep. Serotonin, an inhibitory neurotransmitter, exhibits reduced release during this stage. The decrease in serotoninergic activity contributes to the loss of muscle tone and REM atonia observed during REM sleep, preventing the enactment of dream content. GABAergic neurons also play a role in the transition to REM sleep. The ventrolateral periaqueductal gray (vlPAG) and the lateral pontine tegmentum contain GABAergic neurons that inhibit the activity of REM-on neurons in the SLD and vPAG during NREM sleep. As NREM sleep progresses, the inhibition is gradually lifted, allowing REM-on neurons to become more active [41,42,43,44].

The balance between excitatory and inhibitory inputs within these neural circuits determines the initiation and maintenance of REM sleep, a state characterized by heightened brain activity, dreaming, and muscle atonia.

### 2.4. Sleep, Inflammation and Stress

Sleep has profound effects on the immune system, influencing the production and activity of inflammatory mediators. During NREM sleep, particularly SWS, there is an upregulation of anti-inflammatory cytokines, such as interleukin-10 (IL-10), and a downregulation of pro-inflammatory cytokines, including tumor necrosis factor-alpha (TNF-α) and interleukin-6 (IL-6). The shift towards an anti-inflammatory state during sleep is thought to facilitate tissue repair and regeneration. Conversely, inflammation can modulate sleep patterns. Pro-inflammatory cytokines, such as TNF-α and IL-1β, are known to promote sleep by acting on sleep-regulating regions in the CNS. These cytokines influence the activity of sleep-promoting neurons, including those in the VLPO. In conditions of acute or chronic inflammation, there is often a disruption of sleep architecture, characterized by increased wakefulness and reduced SWS [45,46].

The role of neurotransmitters in the sleep–inflammation interplay is significant. For example, serotonin derived from the raphe nuclei in the brainstem promotes SWS, and its deficiency is associated with sleep disturbances. Serotonin also exerts anti-inflammatory effects by inhibiting the production of pro-inflammatory cytokines [47,48]. 

The molecular mechanisms of the sleep–inflammation relationship extend to the peripheral immune system. The circadian rhythm influences immune cell trafficking, cytokine production, and the activity of immune effector cells. Disturbed sleep patterns, as seen in conditions like sleep apnea or insomnia, are associated with an increased risk of systemic inflammation and immune dysregulation [46,49].

Moreover, the sympathetic nervous system (SNS) and the hypothalamic-pituitary-adrenal (HPA) axis are crucial players in the sleep–inflammation axis. Activation of the SNS and release of stress hormones, such as cortisol, can modulate immune responses and contribute to systemic inflammation. Dysregulation of HPA axis activity can lead to a cascade of sleep disturbances, including insomnia, sleep fragmentation, and alterations in sleep architecture. As a central player in orchestrating the body’s stress response, the HPA axis has the ability to disrupt the finely tuned equilibrium of the circadian rhythm and impact various elements affecting the SWC. Cortisol typically follows a diurnal pattern, peaking in the morning and tapering off at night. However, dysregulated HPA axis activity can disrupt this rhythm, leading to untimely surges in cortisol levels, particularly during the evening and nighttime hours [50,51,52]. 

These irregular cortisol levels can have a detrimental impact on the GABAergic system, a crucial inhibitory neural network. Reduced GABAergic inhibition can result in heightened neural excitability, making the initiation and maintenance of sleep challenging. Additionally, the HPA axis engages in a complex interplay with circadian clock genes like PER1 and PER2. Dysregulated cortisol release can disturb the normal expression of these genes, causing imbalances in the body’s intrinsic chronometer and the precise timing of sleep–wake cycles. This discord can undermine the pineal gland’s ability to produce melatonin, disrupting the hormone’s typical surge in the evening, which signals the body to initiate sleep. The repercussions of dysregulated cortisol extend beyond hormonal fluctuations to the autonomic nervous system. Increased sympathetic nervous system activity, a characteristic of the body’s fight-or-flight response, sustains a state of heightened alertness and readiness to deal with stressors, making relaxation and sleep initiation challenging. Furthermore, the hippocampus, a region involved in memory and stress regulation, contains numerous glucocorticoid receptors. Altered cortisol function can impair hippocampal performance, negatively affecting sleep quality and memory consolidation. Additionally, it contributes to the increase in proinflammatory cytokines, known for disrupting sleep patterns and promoting insomnia [53,54,55].

## 3. Glial Cells: The Orchestrators of Neural Symphony

Glial cells, comprising astrocytes, oligodendrocytes, and microglia, constitute a significant portion of the neural milieu. Long overshadowed by their more excitable counterparts, neurons, glial cells have emerged as dynamic players, orchestrating a myriad of functions essential for the health and function of the nervous system [56].

**Figure 1:**
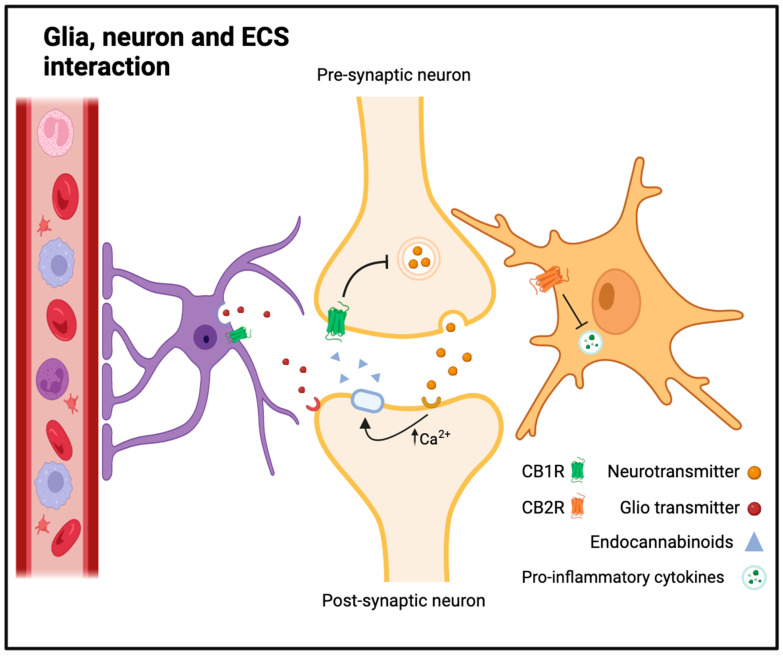
Schematic representation of glia, neuron and ECS interaction. Within the neuron, phospholipids in the membrane serve as precursors for the on-demand synthesis of endocannabinoids, specifically 2-arachidonoylglycerol (2-AG) and Anandamide (AEA). This synthesis occurs in response to physiological demands, enabling the neurons to modulate their signaling. The presynaptic terminals of neurons feature CB1 receptors (CB1R). Upon activation, CB1R negatively modulates calcium influx, leading to a reduction in neurotransmitter release. This negative feedback mechanism acts as a regulatory element in neuronal signaling, finely tuning synaptic transmission. Astrocytes, in close proximity to neurons, play a pivotal role in bidirectional communication. They have the capacity to express CB1R. Stimulation of CB1R in astrocytes positively modulates calcium influx and enhances the release of glutamate, contributing to the intricate balance in cellular communication between astrocytes and neurons. Microglia, the immune cells of the central nervous system, express CB2 receptors (CB2R). Activation of CB2R in microglia exerts a negative modulation on the release of pro-inflammatory cytokines, specifically Tumor Necrosis Factor-alpha (TNFα) and Interleukins (ILs). This signifies the anti-inflammatory effects of the ECS in the immune response, emphasizing its role in maintaining neural tissue homeostasis. All figures were created in BioRender.

Astrocytes, the star-shaped glial cells, are integral to the regulation of extracellular ion concentrations and neurotransmitter levels. Their fine processes ensheath synapses, forming the “tripartite synapse” where they modulate synaptic transmission (Figure 1). By regulating the balance of ions and neurotransmitters, astrocytes contribute to the maintenance of neural homeostasis, ensuring optimal conditions for signaling and preventing excitotoxicity. Oligodendrocytes, responsible for myelinating axons in the central nervous system, play a crucial role in facilitating rapid and efficient signal transmission. Myelin sheaths not only insulate axons but also provide metabolic support to neurons. Disruptions in myelination processes, as observed in demyelinating disorders like multiple sclerosis, highlight the indispensability of oligodendrocytes for neural function. Microglia, the resident immune cells of the central nervous system, are instrumental in surveilling and responding to changes in the neural environment. These cells actively participate in immune responses, clearing cellular debris, and modulating inflammation. The delicate balance between microglial activation and suppression is crucial for preventing excessive inflammation, which can have detrimental effects on neuronal health [56,57,58].

### 3.1. Glial Involvement in Synaptic Plasticity

Recent research has unveiled the active participation of glial cells in synaptic plasticity, the fundamental process underlying learning and memory. Two major types of glial cells, astrocytes and microglia, actively contribute to the regulation of synaptic strength through a range of molecular processes. 

Astrocytes, once considered passive, have emerged as active modulators of synaptic plasticity, participating in both signal integration and modulation of neurotransmission. Neuronal activity triggers an increase in intracellular calcium levels in astrocytes. This calcium signaling is pivotal for the subsequent involvement of astrocytes in synaptic plasticity (Figure 1). In response to elevated calcium, astrocytes release gliotransmitters, including glutamate and D-serine. The released glutamate can bind to presynaptic metabotropic glutamate receptors (mGluRs), inhibiting further neurotransmitter release. Additionally, astrocytic glutamate activates postsynaptic N-methyl-D-aspartate receptors (NMDARs), contributing to long-term potentiation (LTP) at glutamatergic synapses. Astrocytes also release D-serine, a co-agonist of NMDARs, enhancing their function. This modulates NMDAR activity and plays a critical role in synaptic plasticity, particularly LTP. In addition, astrocytes actively participate in clearing excess synaptic glutamate through transporters. This uptake mechanism helps prevent spillover and maintains the balance of neurotransmitters in the synaptic cleft [59,60,61,62].

Interestingly, microglia, traditionally recognized as immune cells, play active roles in synaptic plasticity, contributing to the refinement of neural circuits. Microglia are involved in the elimination of weak or inactive synapses through a process known as synaptic pruning. This contributes to the refinement and optimization of synaptic connections. Activated microglia release signaling molecules such as cytokines and neurotrophic factors. These molecules can modulate synaptic function, with brain-derived neurotrophic factor (BDNF) influencing synaptic strength. On the other hand, microglia also actively phagocytose synapses, a process referred to as synaptic stripping. This phagocytic activity contributes to the removal of unwanted or damaged synapses, shaping synaptic connectivity [63,64].

### 3.2. Glial Involvement in the Removal of Waste Products and Brain Homeostasis

**Figure 2:**
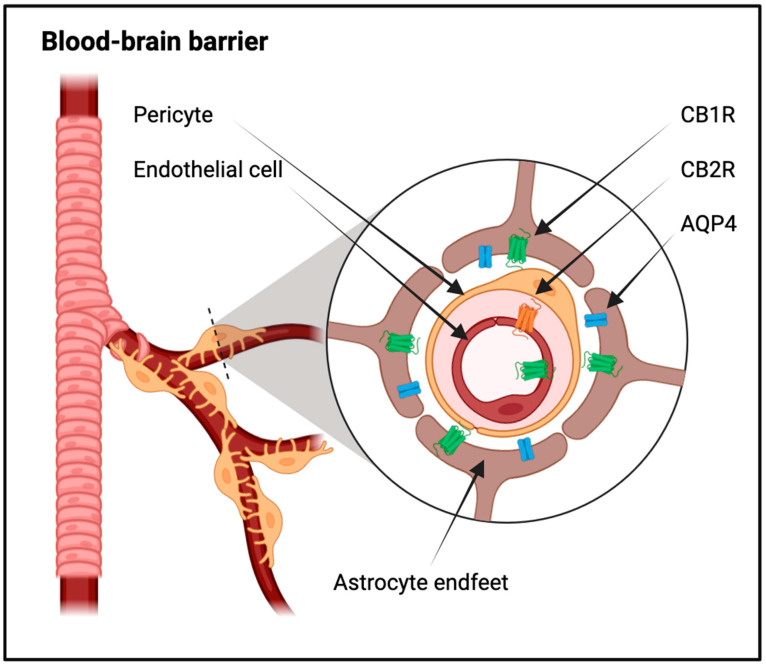
Schematic representation of the blood–brain barrier. The Blood–Brain Barrier (BBB) is a pivotal interface regulating molecular exchange between the bloodstream and the brain parenchyma. The blood vessel, portrayed as a capillary, is defined by endothelial cells forming its walls. The endothelial cells are accentuated to underscore the presence of tight junctions, crucial components restricting the free movement of macromolecules. These tight junctions between adjacent endothelial cells constitute the primary foundation of the BBB, ensuring a highly selective barrier. BBB endothelial cells are further highlighted, with an emphasis on the luminal and abluminal sides. On the luminal side, CB1 receptors (CB1R) are depicted, illustrating their strategic location facing the bloodstream. Conversely, CB2 receptors (CB2R) are situated on the abluminal side, engaging with the brain parenchyma. This spatial distribution of cannabinoid receptors signifies their distinct roles in modulating molecular transport and signaling processes at the BBB. The perivascular space, a microenvironment situated between endothelial cells, is portrayed as a conduit for controlled diffusion. This space allows fluid and solutes to move from the bloodstream into the brain parenchyma, contributing to the dynamic exchange of substances. Astrocytic endfeet extend from astrocytes toward the blood vessel, forming a close association with endothelial cells. These endfeet express Aquaporin-4 (AQP4) channels, facilitating water transport, and CB1 receptors (CB1R), indicating their active involvement in regulatory processes. This sophisticated interaction between endothelial cells and astrocytic endfeet showcases the collaborative efforts in maintaining BBB integrity.

The brain is a highly metabolically active organ that generates substantial waste products that require efficient clearance mechanisms. The glymphatic system is a recently uncovered mechanism in the brain that relies on the active participation of glial cells, particularly astrocytes, to facilitate the efficient removal of waste products and maintain the brain’s homeostasis. A crucial component of this system is the water channel protein Aquaporin-4 (AQP4), predominantly expressed on astrocytic endfeet surrounding blood vessels. These endfeet create a structural framework enabling the convective flow of cerebrospinal fluid (CSF) into the brain parenchyma, allowing for the exchange of water and solutes between the CSF and interstitial fluid (Figure 2) [65,66].

The glymphatic system functions by influxing CSF into the brain parenchyma via perivascular spaces surrounding arteries. This convective flow assists in flushing out waste products, including soluble proteins and metabolic byproducts, from the interstitial space. Astrocytes, marked by the expression of glial fibrillary acidic protein (GFAP), play a pivotal role in the regulation of the glymphatic system. The dynamic regulation of GFAP is associated with changes in AQP4 expression, influencing the efficiency of waste clearance in different physiological and pathological conditions.

A significant aspect of the glymphatic system is its sleep-dependent activation, with increased activity observed during NREM 3. The expansion of the interstitial space during sleep enhances the convective flow of CSF, facilitating the efficient removal of waste products. Neurotransmitters, such as norepinephrine, have been implicated in the regulation of glymphatic function, as their activation of specific receptors can modulate astrocytic activity and AQP4 expression, influencing the system’s efficiency [67,68].

The glymphatic system’s effectiveness may decline with age, contributing to the increased vulnerability to neurodegenerative diseases. Conditions like Alzheimer’s disease are associated with impaired glymphatic function and the accumulation of protein aggregates [69,70].

### 3.3. Glial Cells in the Complex Tapestry of Neuroinflammation

Neuroinflammation, a complex and dynamic response in the CNS, involves the interaction of glial cells, primarily microglia and astrocytes, in a finely regulated molecular relationship. 

Microglia, as the resident immune cells of the CNS, play a central role in the initiation and propagation of neuroinflammatory responses. When activated by various stimuli such as pathogens, injury, or abnormal protein aggregates, microglia undergo morphological changes and shift to an activated state. This activation involves the recognition of danger signals through pattern recognition receptors (PRRs), including Toll-like receptors (TLRs) and Nod-like receptors (NLRs). The engagement of these receptors triggers downstream signaling cascades, leading to the activation of nuclear factor-kappa B (NF-κB) and mitogen-activated protein kinase (MAPK) pathways, ultimately resulting in the expression of pro-inflammatory cytokines such as interleukin-1 beta (IL-1β), tumor necrosis factor-alpha (TNF-α), and interleukin-6 (IL-6) [64,71,72].

Astrocytes, the most abundant glial cells in the CNS, are also active participants in neuroinflammation. They respond to inflammatory signals by upregulating various inflammatory mediators and cytokines. Importantly, astrocytes contribute to the maintenance of the blood–brain barrier (BBB) and regulate the influx of immune cells into the CNS (Figure 1 and Figure 2). In the context of neuroinflammation, astrocytes undergo astrogliosis, a process characterized by hypertrophy and increased expression of GFAP [59,73,74].

The crosstalk between microglia and astrocytes is a critical aspect of neuroinflammation modulation. Microglia release signaling molecules such as ATP, which can activate purinergic receptors on astrocytes, triggering their reactive responses. Conversely, activated astrocytes release various factors, including cytokines and chemokines, that can influence microglial activity. This bidirectional communication amplifies the inflammatory response and contributes to the perpetuation of neuroinflammation [75].

In addition to pro-inflammatory mediators, glial cells are also involved in the production of anti-inflammatory molecules. The release of transforming growth factor-beta (TGF-β) and interleukin-10 (IL-10) by both microglia and astrocytes serves as a counterbalance to the pro-inflammatory milieu, promoting resolution and tissue repair. The activation of specific receptors, such as the TGF-β receptor, initiates downstream signaling pathways that suppress inflammation and promote an anti-inflammatory microenvironment. Moreover, glial cells actively participate in the clearance of debris and damaged cells through phagocytosis, a crucial process for resolving inflammation and preventing chronicity. Microglia, in particular, are adept phagocytes and contribute to the removal of apoptotic neurons, protein aggregates, and other cellular debris [76,77,78,79,80,81]. The molecular mechanisms underlying the glial modulation of neuroinflammation also involve epigenetic regulation. Changes in DNA methylation, histone modifications, and microRNA expression patterns can influence the inflammatory phenotype of both microglia and astrocytes. These epigenetic modifications contribute to the long-lasting effects of neuroinflammation and may influence the susceptibility to chronic neurodegenerative diseases [82,83].

## 4. Glial Orchestration of Sleep

The regulation of sleep, a fundamental physiological process, involves not only the well-studied neural networks but also the active participation of glial cells. Glial cells, once considered passive bystanders, are emerging as key players in the orchestration of the SWC. 

Astrocytes, the most abundant glial cells in the brain, play a pivotal role in maintaining the homeostasis critical for sleep. Through their fine processes enveloping synapses, astrocytes actively regulate neurotransmitter levels, ensuring the delicate balance essential for normal sleep–wake transitions. The tripartite synapse, formed by astrocytes, neurons, and synapses, highlights the dynamic communication necessary for optimizing neural signaling during sleep. The involvement of astrocytes in the clearance of adenosine, a sleep-promoting molecule, underscores their role in the homeostatic drive for sleep. Additionally, astrocytes release signaling molecules such as D-serine, influencing sleep-related processes by NMDA receptor activity [84,85].

Microglia extend their influence on sleep regulation through intricate immunomodulatory mechanisms. The bidirectional relationship between sleep and immune function is increasingly recognized, and microglia play a crucial role in this interplay. During sleep, microglia engage in surveillance and the removal of cellular debris, contributing to the overall maintenance of neural health. Conversely, sleep disturbances can activate microglia, leading to neuroinflammation. Chronic activation of microglia has been implicated in sleep disorders and cognitive impairments. The dynamic modulation of microglial function during sleep suggests a critical role in the bidirectional communication between the immune system and sleep regulatory pathways [86,87].

Oligodendrocytes, traditionally associated with myelination, are increasingly recognized for their contributions to sleep-related processes. Myelin, the insulating sheath surrounding axons, facilitates rapid signal transmission. Recent studies have suggested that oligodendrocytes play a role in modulating the timing and efficiency of sleep-related neural circuits. Oligodendrocytes express a range of receptors and ion channels that respond to neurotransmitters and neuromodulators implicated in sleep regulation. For instance, they express receptors for GABA. GABAergic signaling is crucial for promoting sleep, and oligodendrocytes respond to GABA by adjusting their membrane potential and ion conductance, potentially influencing the excitability of surrounding neurons. 

Moreover, oligodendrocytes express receptors for adenosine, a nucleoside that accumulates during wakefulness and promotes sleep. Adenosine receptors on oligodendrocytes may participate in the homeostatic regulation of sleep by responding to the adenosine-mediated reduction in neural activity and contributing to the modulation of myelin-related processes [88,89]. Intriguingly, oligodendrocytes exhibit circadian rhythmicity in their gene expression, suggesting that they are under the influence of the central circadian clock, which is regulated by the SCN. This circadian regulation may extend to the timing of myelination and the modulation of sleep-related neural circuits. Furthermore, oligodendrocytes release extracellular vesicles containing microRNAs that can influence the expression of genes in neighboring cells. These microRNAs may contribute to the dynamic communication between oligodendrocytes and neurons, potentially impacting the timing and efficiency of sleep-related neural circuits [90,91].

The interaction between oligodendrocytes and sleep-related circuits is likely bidirectional. Neuronal activity, influenced by the SWC, can impact oligodendrocyte function and myelination. In turn, the myelination status of axons can affect the speed and reliability of neural signal transmission, influencing the overall efficiency of sleep-related circuits. Disruptions in oligodendrocyte function and myelination have been implicated in sleep disorders and neurological conditions associated with altered sleep patterns. The plasticity of myelin, influenced by sleep patterns, also raises intriguing questions about the role of oligodendrocytes in sleep-dependent memory consolidation. 

## 5. Dysfunction of the Glial System and Its Association with Sleep Disorders

Sleep disorders encompass a spectrum of conditions ranging from insomnia to sleep-related breathing disorders, parasomnias, and circadian rhythm disorders. These conditions are complex and multifaceted, and significantly impact the quality of life of affected individuals. Recent advancements in neuroscience have highlighted the critical involvement of glial cells, including astrocytes and microglia, in the regulation of sleep–wake cycles. 

### 5.1. Insomnia

Insomnia, characterized by persistent difficulties in falling or staying asleep, is intricately linked to alterations in glial function within the CNS. Glial cells, particularly astrocytes and microglia, play pivotal roles in maintaining neural homeostasis and regulating synaptic activity [92,93].

Astrocytes contribute to the regulation of neurotransmitters, such as glutamate. Disruption in astrocytic function may lead to an imbalance in glutamate levels, affecting synaptic transmission. This disturbance in neurotransmitter homeostasis can influence the overall excitatory–inhibitory equilibrium, potentially contributing to difficulties in transitioning between wakefulness and sleep. Furthermore, astrocytes are involved in the release of gliotransmitters, which modulate synaptic plasticity. Dysregulation of these gliotransmitter signals can impact the functioning of neural circuits involved in sleep regulation, adding another layer to the potential pathophysiology of insomnia [94,95].

Microglia also play a crucial role in conditions like insomnia; microglia may become activated, leading to neuroinflammation. Activated microglia release proinflammatory cytokines and other signaling molecules, creating an inflammatory environment. This neuroinflammatory response can contribute to the hyperarousal state observed in insomnia, disrupting the normal sleep–wake cycle [94].

### 5.2. Sleep-Related Breathing Disorders

These disorders encompass a range of conditions, including sleep apnea, characterized by interruptions in breathing during sleep. These involve complex interactions within the respiratory control centers, neuromuscular pathways, and the regulation of upper airway muscles. In sleep apnea, the dysfunction of glial cells, particularly astrocytes and microglia, may contribute to altered neural signaling and respiratory control. Disruption in astrocytic function could impact the regulation of neurotransmitters involved in respiratory control, influencing the coordination of breathing patterns during sleep, whereas microglial activation may occur due to intermittent hypoxia and other stressors associated with disrupted breathing. Activated microglia can release proinflammatory mediators, contributing to neuroinflammation and potentially affecting the neural circuits involved in respiratory regulation. Moreover, the dysregulation of glial cells in the context of sleep-related breathing disorders may impact the neuromuscular control of upper airway muscles. This could result in an increased propensity for airway collapse or obstruction during sleep, a hallmark of sleep apnea [95,96,97,98].

### 5.3. Parasomnias

Parasomnias represent a group of sleep disorders characterized by abnormal behaviors, movements, emotions, perceptions, or dreams that occur during different stages of sleep. The alterations behind these disorders involve intricate interactions within the neural circuits, neurotransmitter systems, and glial functions that govern sleep architecture and transitions between sleep stages. The dysregulation of glial functions may contribute to disruptions in sleep-related neural circuits and aberrant behaviors during sleep. Astrocytes, with their involvement in neurotransmitter clearance and synaptic modulation, could influence the balance of excitatory and inhibitory neurotransmitters critical for the regulation of sleep–wake transitions. Dysfunction in astrocytic processes might lead to imbalances in neurotransmitter levels, impacting the stability of sleep stages and contributing to parasomnias. Also, alterations in microglial activation may lead to localized neuroinflammatory responses, affecting the integrity of sleep-related neural networks and potentially triggering abnormal behaviors during sleep [99,100,101].

Furthermore, the interaction between glial cells and the glymphatic system, responsible for waste clearance from the brain during sleep, could be relevant. Disruptions in glial-mediated clearance mechanisms might contribute to the accumulation of substances that influence sleep-related neural circuits, potentially leading to parasomnias.

### 5.4. Circadian Rhythm Disorders

These occur when the natural sleep–wake cycle is disrupted, leading to difficulties in falling asleep, staying asleep, or experiencing alertness at appropriate times. The molecular and cellular basis for circadian rhythm disorders involves a complex network of interactions between the central circadian clock, peripheral tissues, neurotransmitter systems, and glial functions. At the core of circadian rhythm regulation is the central circadian clock located in the SCN of the hypothalamus. Glial cells, particularly astrocytes, interact with the SCN and may influence the expression of clock genes. Dysregulation of glial functions could impact the molecular machinery responsible for maintaining circadian rhythmicity. Astrocytes, through their fine processes ensheathing synapses, may modulate neurotransmitter release in areas relevant to circadian rhythm regulation. Dysfunctional astrocytic regulation of neurotransmission might disrupt the finely tuned balance between wakefulness and sleep, contributing to circadian rhythm disorders. Microglial activation in response to inflammation or other signals might influence the overall homeostasis of the circadian system, potentially contributing to disruptions in sleep–wake patterns. Additionally, the interaction between glial cells and the endocrine system, including the pineal gland and melatonin production, is critical for circadian rhythm regulation. Dysfunction in glial-mediated processes may impact the release and regulation of melatonin, a key hormone involved in sleep–wake cycles. Moreover, glial cells are implicated in the modulation of synaptic plasticity, which is crucial for the adaptation of neural circuits to changing environmental conditions, including light–dark cycles [81,102]. 

Dysregulation in glial-mediated synaptic plasticity might contribute to circadian rhythm disorders by disrupting the ability of neural circuits to synchronize with the external environment.

### 5.5. Restless Legs Syndrome 

Restless Legs Syndrome (RLS) is a neurological disorder characterized by an irresistible urge to move the legs, often accompanied by uncomfortable sensations. Iron deficiency in the brain, particularly in the substantia nigra, has been implicated in the pathophysiology of RLS. Glial cells, such as astrocytes and microglia, play crucial roles in iron homeostasis. Dysregulation of glial iron-handling processes may contribute to alterations in neural function and neurotransmission, potentially triggering RLS symptoms. Dysfunctional astrocytic processes may disrupt the balance of neurotransmitters such as dopamine, which is implicated in motor control and has been associated with RLS. Furthermore, hormones such as cortisol and sex hormones may modulate RLS symptoms. Dysregulation in glial-mediated hormonal responses could impact the manifestation of RLS symptoms, particularly in relation to circadian variations [103,104].

### 5.6. Narcolepsy

Narcolepsy is characterized by the loss of hypocretin-producing neurons and dysregulation of neurotransmitter systems [105,106]. In the context of narcolepsy, dysregulation of astrocytic functions emerges as a contributing factor to the observed neuroinflammation. Dysfunctional astrocytes may disrupt glial transmission and impair synaptic regulation, fostering an inflammatory milieu within the CNS. Also, dysfunction in astrocytic processes may contribute to the reduction of hypocretin levels, leading to a deficit in this wake-promoting neuropeptide. Autoimmune mechanisms result in the destruction of hypocretin-producing neurons, triggering microglial activation. Activated microglia release pro-inflammatory cytokines, contributing to the inflammatory environment within the CNS [107]. Astrocytes also contribute significantly to the maintenance of the BBB. Through their interactions with endothelial cells, astrocytes regulate the exchange of substances between the blood and the brain. Dysfunction in astrocyte–endothelial interactions may compromise the integrity of the BBB in narcolepsy, allowing immune cells and inflammatory mediators to penetrate the CNS. This breach in the BBB further contributes to the neuroinflammatory processes associated with narcolepsy.

## 6. The Endocannabinoid System

Comprising endogenous cannabinoids, receptors, and enzymes, the ECS modulates various physiological processes, including mood, appetite, sleep, immune response, and pain sensation. The primary endocannabinoids are anandamide (AEA) and 2-arachidonoylglycerol (2-AG). AEA, named after the Sanskrit word for bliss, and 2-AG are lipid molecules synthesized on demand in response to cellular needs. These endocannabinoids act as retrograde messengers, traveling backward across synapses to bind to cannabinoid receptors on presynaptic neurons. This retrograde signaling mechanism allows the ECS to regulate neurotransmitter release and synaptic plasticity [108,109].

Cannabinoid receptors, CB1 and CB2 receptors (CB1R and CB2R, respectively), are G protein-coupled receptors found throughout the body. CB1R is predominantly located in the central nervous system, particularly in the brain regions associated with mood, cognition, and pain perception. CB2R is mainly found in immune cells and peripheral tissues (Figure 1). When endocannabinoids bind to these receptors, they activate intracellular signaling pathways that modulate cellular functions. Upon binding to ligands, activated cannabinoid receptors engage with and activate specific G proteins situated within the cell. CB1R predominantly triggers Gαi/o proteins, whereas CB2R can associate with both Gαi/o and Gαs proteins. The activated G proteins undergo subunit dissociation, influencing intracellular signaling pathways. In the case of CB1R, Gαi/o inhibits cyclic adenosine monophosphate (cAMP) production and diminishes calcium ion influx into neurons, thereby regulating neurotransmitter release. CB2R activation also inhibits cAMP production but stimulates other signaling pathways related to immune responses and inflammation, such as the MAP kinase phosphatase 3 (MKP3) pathway. Cannabinoid receptors primarily function to regulate the release of various chemical messengers. CB1R modulates the release of specific neurotransmitters, safeguarding the CNS against excessive stimulation or inhibition induced by other neurotransmitters. Conversely, CB2R primarily assumes a peripheral role with immunomodulatory activities, chiefly regulating the release of cytokines [108,109,110,111]. Consequently, cannabinoids may influence neurodegenerative diseases through their neuro- and immunomodulatory effects [112].

The enzymes responsible for the synthesis and degradation of endocannabinoids are crucial in regulating their levels. Two main enzymes involved in this process are fatty acid amide hydrolase (FAAH) and monoacylglycerol lipase (MAGL). FAAH breaks down AEA, while MAGL degrades 2-AG. These enzymes ensure the timely termination of endocannabinoid signaling, preventing excessive activation of cannabinoid receptors [113].

The ECS is involved in various physiological processes, and its modulation is often context-dependent. For example, in the central nervous system, the ECS plays a neuromodulatory role, regulating neurotransmitter release. AEA and 2-AG act on CB1R to inhibit the release of neurotransmitters like GABA and glutamate. This modulation contributes to the fine-tuning of synaptic transmission and influences processes such as memory, mood, and pain perception. Beyond the central nervous system, the ECS has widespread effects on peripheral tissues. CB2R in immune cells play a role in regulating inflammation and immune responses. Activation of CB2R can modulate the release of cytokines and other immune signaling molecules, impacting the immune system’s ability to maintain balance and respond to challenges [113,114] (Figure 1).

The ECS is also implicated in various pathological conditions, and its pharmacological modulation has therapeutic potential. Exogenous cannabinoids, such as those found in the Cannabis plant, can interact with the ECS, leading to therapeutic effects. Cannabidiol (CBD), for example, is a non-psychoactive compound derived from cannabis that interacts with CB1 and CB2R, exerting anti-inflammatory, analgesic, and anxiolytic effects [74]. The ECS orchestrates a wide array of physiological functions, reflecting its ubiquitous presence in the body. In the central nervous system, CB1R modulate neurotransmitter release, impacting mood, memory, and pain perception. Peripheral tissues, particularly those associated with the immune system, are regulated by CB2R, influencing inflammation and immune responses [115,116,117].

The ECS plays a vital role in maintaining homeostasis, ensuring that internal conditions remain within a stable range. It regulates processes such as appetite, sleep, and stress responses. 

### 6.1. Endocannabinoids and the Sleep–Wake Cycle

The endogenous ligands of the ECS, mainly AEA and 2-AG, play a pivotal role in the modulation of sleep. These are synthesized on demand in response to various stimuli, including changes in sleep patterns. The concentrations of these molecules fluctuate across the sleep–wake cycle, suggesting their involvement in the regulation of sleep stages. Studies have demonstrated that AEA levels increase during NREM sleep, particularly during the SWS phase, indicating a potential role in the restorative aspects of sleep. Conversely, 2-AG levels exhibit a surge during REM sleep, implicating its involvement in the modulation of dreaming and cognitive processes associated with this sleep stage [118,119].

The ECS exerts its influence on sleep through the activation of cannabinoid receptors, primarily CB1R and CB2R. CB1R are abundant in the central nervous system, with particularly high densities in regions associated with sleep regulation, such as the hypothalamus and brainstem. CB2R, found predominantly in immune cells, also contribute to the neuroimmune modulation of sleep. The ECS, through the activation of CB1R, modulates the release of neurotransmitters associated with the sleep–wake cycle. The ECS’s influence on GABAergic and glutamatergic neurotransmission holds particular significance. The ECS, by acting on CB1R, enhances GABAergic signaling, contributing to the induction of sleep and the maintenance of NREM sleep stages. Conversely, the modulation of glutamate release by the ECS impacts wakefulness, illustrating the dual role of this system in fine-tuning the delicate balance between sleep and wakefulness [120,121].

Within the intricate nuclei of the brain’s sleep-regulating centers, the ECS actively engages with the mechanisms that shape the architecture of sleep. Notably, studies suggest that the activation of CB1R is associated with an augmentation of slow-wave sleep, the restorative phase of NREM sleep characterized by synchronized neural activity and reduced sensory input. Moreover, the ECS plays a role in responding to external factors influencing the sleep–wake cycle. Stress, a potent disruptor of sleep patterns, induces changes in endocannabinoid levels, reflecting the ECS’s adaptive response to maintain homeostasis. The circadian rhythm, governed by the SCN, also intertwines with the ECS, influencing the temporal release of endocannabinoids and further emphasizing its role in circadian sleep regulation [121,122].

The ECS and adenosine signaling are intricately linked, adding another layer of complexity to sleep modulation. CB1R, expressed in various brain regions, including those involved in adenosine-mediated effects, influence adenosine receptor signaling. Activation of CB1R has been shown to modulate adenosine release and adenosine receptor sensitivity. This interaction suggests that the ECS contributes to the fine-tuning of adenosinergic signaling, influencing the homeostatic drive for sleep [123]. Moreover, the role of adenosine in sleep is further highlighted by the action of caffeine. Caffeine, a well-known adenosine receptor antagonist, counteracts the sedative effects of adenosine, providing temporary relief from sleepiness [124]. This interaction also implies a potential interplay between the ECS and the effects of caffeine on adenosine receptors.

The finely tuned regulation of endocannabinoid levels relies on the enzymatic machinery of the ECS, specifically fatty acid amide hydrolase (FAAH) and monoacylglycerol lipase (MAGL). FAAH primarily breaks down AEA, while MAGL is responsible for degrading 2-AG. The dynamic activity of these enzymes influences the temporal release and degradation of endocannabinoids, contributing to the modulation of sleep. Studies have shown that inhibiting these enzymes leads to an accumulation of endocannabinoids, promoting sleep and enhancing sleep continuity [72,74,75]. This suggests that targeting FAAH and MAGL may hold therapeutic potential for sleep-related disorders by extending the presence of endocannabinoids during critical phases of the SWC [113,125].

### 6.2. Endocannabinoid Modulation of Immune Function and Inflammation

The ECS oversees the regulation of immune function, with a particular emphasis on CB2R, which are abundant in various immune cells. These receptors are predominantly expressed in immune cells and play a crucial role in modulating the body’s immune responses. The interaction between the ECS and immune function is of significant scientific interest due to its potential implications for comprehending and addressing conditions marked by immune dysregulation. CB2R are widespread in diverse immune cells, including leukocytes, monocytes, macrophages, B cells, T cells, and natural killer cells. Their presence in these cells underscores the importance of CB2R in governing immune responses. While CB2R are most prevalent in immune cells, they are also found in immune-associated tissues such as the spleen and tonsils. Additionally, they are expressed in the bone marrow, a central site for immune cell production. As mentioned previously, CB2R activation predominantly triggers Gαi/o and Gαs proteins. As CB2R becomes activated, it inhibits adenylyl cyclase activity, responsible for converting ATP to cyclic AMP (cAMP). The reduction in cAMP levels has downstream effects, influencing ion channel activity, particularly the inhibition of calcium channels and the activation of potassium channels. These changes in ion channel dynamics contribute to alterations in membrane potential [111,112].

Simultaneously, CB2R activation initiates signaling through the MAPK pathway. MAPKs, such as extracellular signal-regulated kinases 1 and 2 (ERK1/2), become activated, influencing various cellular responses. Notably, CB2R activation orchestrates the inhibition of the nuclear factor-kappa B (NF-κB) pathway. NF-κB is a transcription factor central to the expression of proinflammatory genes. By inhibiting NF-κB, CB2R helps decrease the transcription of proinflammatory cytokines, crucial players in the inflammatory cascade. CB2R activation also prompts the release of anti-inflammatory mediators, such as IL-10. IL-10, renowned for its anti-inflammatory properties, aids in resolving immune responses and maintaining immune homeostasis. CB2R further modulates the release of inflammatory mediators like TNF-α and IL-1β. This modulation contributes to the overall dampening of the inflammatory response. Interestingly, CB2R activation has been associated with anti-apoptotic effects, enhancing cell survival in certain contexts. This adds another layer to the multifaceted role of CB2 receptors in cellular homeostasis. Additionally, CB2R activation influences immune cell migration and chemotaxis, impacting the recruitment of immune cells to sites of inflammation [126,127,128,129].

The ECS plays a pivotal role in maintaining immune homeostasis, ensuring that the immune system responds appropriately to threats while preventing excessive or chronic inflammation. This immunomodulatory function contributes to sustaining a balanced and regulated immune response. This interaction is achieved by inhibiting the ERK pathway through MAPK induction. The ECS’s ability to regulate immune responses and inflammation at the molecular level opens potential avenues for therapeutic interventions. 

## 7. ECS–Glia Axis: Molecular Mechanisms and Cellular Interactions 

The bidirectional communication within the ECS–Glia Axis intersects with sleep regulation, creating a dynamic relationship between neuroinflammation and sleep–wake patterns. Sleep disturbances often coincide with increased neuroinflammation, and chronic activation of the ECS–Glia Axis may contribute to disrupted sleep. Conversely, alterations in sleep architecture influence the activity of the ECS–Glia Axis, suggesting a reciprocal modulation.

### 7.1. Endocannabinoid Regulation of Glial Function

The nervous system, once viewed solely through the lens of neurons, now reveals a complex symphony where glial cells and the ECS emerge as essential players. Endocannabinoid signaling is deeply intertwined with glial cell activity. 

Astrocytes, with their fine processes ensheathing synapses, express both CB1R and CB2R. Activation of CB1R on astrocytes modulates neurotransmitter release and synaptic plasticity, contributing to the fine-tuning of neural networks. Upon activation of CB1R on astrocytes, a cascade of intracellular events is initiated. The primary consequence is the modulation of neurotransmitter release from neighboring neurons. Astrocytes, in response to endocannabinoid signaling, regulate the release of neurotransmitters such as glutamate and GABA. This modulation occurs through the control of calcium signaling and exocytosis machinery within astrocytic processes. The activation of CB1R on astrocytes also exerts profound effects on synaptic plasticity, a fundamental mechanism underlying learning and memory. Astrocytes, through the release of gliotransmitters, influence synaptic strength and contribute to the dynamic regulation of neural circuits. The release of factors such as ATP, D-serine, and prostaglandins by astrocytes modulates the efficacy of synaptic transmission and the induction of LTP or LTD. The orchestrated effects of CB1R activation on astrocytes collectively contribute to the fine-tuning of neural networks. By modulating both neurotransmitter release and synaptic plasticity, astrocytes actively shape the dynamics of neural circuits. This fine-tuning is essential for maintaining homeostasis in the CNS, optimizing signal transmission, and ensuring the appropriate processing of information within neural networks. While CB1R dominate the discussion of astrocytic modulation, the expression of CB2R on astrocytes introduces an additional layer of complexity. CB2R, traditionally associated with immune modulation, may play a role in astrocytic responses to inflammatory stimuli or pathological conditions. Further research is needed to elucidate the specific contributions of CB2R in astrocytic function and their potential impact on neural network dynamics [130,131,132,133,134] (Figure 2).

Microglia, the immune cells of the CNS, express CB2R, suggesting a role for endocannabinoids in immune modulation. In response to neuronal injury or inflammation, microglia become activated, and the ECS acts as a regulator of this immune response, influencing the release of pro-inflammatory and anti-inflammatory mediators (Figure 2). Upon activation in response to neuronal injury or inflammation, microglia undergo morphological and functional changes, transitioning to an activated state. The activation of CB2R on microglia serves as a crucial regulatory mechanism in shaping the ensuing immune response. The ECS, acting through CB2R, modulates the release of pro-inflammatory and anti-inflammatory mediators, influencing the overall immune milieu in the CNS. CB2R activation has been shown to dampen the release of pro-inflammatory cytokines, such as TNF-α and IL-1β, while concurrently promoting the release of anti-inflammatory mediators, including IL-10 and TGF-β. This delicate balance orchestrated by the ECS contributes to the resolution of inflammation and the restoration of neural homeostasis. In situations of neuronal injury or inflammation, microglial activation is a key component of the CNS’s defense and repair mechanisms. The ECS, through CB2R signaling, modulates the intensity and duration of this microglial response. By influencing the release of pro-inflammatory mediators, the ECS contributes to the containment of inflammation, preventing excessive damage to surrounding neurons. Simultaneously, the promotion of anti-inflammatory mediators aids in the resolution of the immune response, fostering an environment conducive to tissue repair [135,136,137,138,139].

Oligodendrocytes, responsible for myelination, also express CB1R. The ECS is implicated in the regulation of myelination processes, influencing the conduction speed of nerve impulses and contributing to the structural integrity of neural circuits. The presence of CB1R on oligodendrocytes has opened a new avenue of investigation into the role of the ECS in myelination processes. CB1R activation on oligodendrocytes modulates intracellular signaling cascades that impact the synthesis and maintenance of myelin. The intricate interplay between endocannabinoid signaling and myelination highlights the adaptability of oligodendrocytes in response to environmental cues and the overall homeostasis of neural circuits. CB1R signaling on oligodendrocytes influences the thickness and compactness of myelin sheaths, directly impacting the conduction speed of nerve impulses. This modulation ensures the rapid and efficient transmission of electrical signals along axons, a critical aspect of neural communication. Beyond its influence on conduction speed, the ECS-mediated regulation of myelination contributes to the overall structural integrity of neural circuits. Oligodendrocyte-derived myelin provides essential support and insulation for axons, preventing signal leakage and maintaining the fidelity of neural communication [140,141,142]. 

### 7.2. Endocannabinoid Regulation of the Central Nervous System Fluid Dynamics

Inflammation within the CNS serves as a pivotal mechanism contributing to the pathogenesis of various neurological conditions. This process unfolds through a complex cascade initiated by inflammatory signals stemming from diverse sources such as infection, injury, or neurodegenerative processes. The orchestrated migration of leukocytes, including neutrophils and monocytes, from the bloodstream across the BBB to the site of inflammation is a crucial aspect of this process. Upon reaching the BBB, leukocytes undergo a series of steps, including initial rolling and subsequent firm adhesion to endothelial cells, followed by transmigration into the CNS tissue [143] (Figure 1). Within the CNS tissue, these migrating leukocytes interact with local immune cells, particularly microglia, triggering the activation of an immune response. This immune response involves the release of pro-inflammatory cytokines, chemokines, and other mediators, aimed at eliminating the source of inflammation. The interaction between circulating leukocytes and resident immune cells can amplify the immune response, intensifying the inflammatory process. Maintaining a balanced resolution of inflammation becomes crucial to prevent tissue damage and the onset of neurodegenerative disorders [144,145].

Inflammatory conditions and neurodegenerative diseases often disrupt the BBB, resulting in increased permeability and compromised brain function. CB1R are primarily located on the luminal side of the BBB endothelium, and their expression has been observed in astrocytes, microglial cells, and pericytes (Figure 2). Activation of CB1R in astrocytes has been linked to the regulation of metabolic activities, enhancing processes crucial for supplying energy to the brain, such as glucose oxidation and ketogenesis. Considering the role of perivascular astrocytes in delivering energy to neurons, the activation of astrocytic CB1R may have implications for regulating the energy supply to neurons [146,147,148,149].

Studies demonstrate that cannabinoids, such as CBD, can modify detrimental effects associated with neuroinflammatory conditions by reducing leukocyte infiltration and down-regulating the expression of inflammatory molecules. CBD has shown the potential to prevent inflammation in endothelial cells, thereby mitigating observed BBB alterations. On the other hand, the activation of CB2R has been associated with decreased surface expression of adhesion molecules, increased tight junction protein levels, and attenuation of BBB damage and neurodegeneration in traumatic brain injury models. Research has further highlighted the positive effects of cannabinoids on BBB integrity and permeability. CBD administration has been linked to enhanced BBB integrity, reduced levels of proinflammatory cytokines, and increased expression of tight junction proteins. The ECS exhibits immunomodulatory properties with potential implications for immune responses within the brain, particularly relevant in the context of neuroinflammatory processes associated with neurological disorders. This interaction gains significance due to its potential consequences for maintaining BBB integrity. Additionally, the ECS’s involvement in neuroprotective functions may indirectly influence BBB performance by counteracting oxidative stress and inflammatory processes, recognized contributors to BBB impairment. In this manner, the ECS might contribute to sustaining BBB integrity and overall cerebral well-being. Furthermore, the ECS exhibits implications in the intricate phenomenon of neurovascular coupling, governing the interplay between neuronal activity, blood flow regulation, and BBB functionality, thereby potentially coordinating neural activity, blood flow dynamics, and BBB functionality [150,151,152,153,154].

### 7.3. Glial Regulation of Endocannabinoid Function

Glial cells actively participate in the regulation of endocannabinoid levels. Astrocytes, through the expression of enzymes like FAAH and MAGL, contribute to the breakdown of AEA and 2-AG, respectively. AEA is known for its modulation of synaptic transmission and involvement in various physiological processes. The expression of FAAH in astrocytes ensures the controlled breakdown of AEA, regulating its spatiotemporal availability within the synaptic cleft. Astrocytic expression of MAGL ensures the regulated breakdown of 2-AG, influencing its concentration and impact on cannabinoid receptors. This enzymatic control over 2-AG levels by astrocytes contributes to the dynamic modulation of ECS signaling. The controlled degradation of AEA and 2-AG by astrocytes is paramount in shaping the spatiotemporal dynamics of endocannabinoid signaling. By regulating the availability of these endocannabinoids, astrocytes exert a precise influence over cannabinoid receptor activation, impacting synaptic transmission and network activity. This fine-tuning ensures that endocannabinoid signaling is dynamic, responsive, and spatially restricted, avoiding unwarranted or prolonged receptor activation [155,156,157].

The meticulous control of endocannabinoid levels by astrocytes has profound functional implications for neural circuitry, synaptic plasticity, and overall brain homeostasis. Dysregulation of ECS signaling is implicated in various neurological disorders, emphasizing the therapeutic potential of targeting astrocytic endocannabinoid enzymes. Strategies that modulate FAAH and MAGL activity in astrocytes hold promise for fine-tuning endocannabinoid levels and mitigating pathological conditions associated with ECS dysfunction. By controlling the degradation of endocannabinoids, astrocytes play a critical role in shaping the spatiotemporal dynamics of endocannabinoid signaling.

### 7.4. Sleep, Synaptic Plasticity, and the Endocannabinoid System in Memory Consolidation 

Sleep is a complex physiological phenomenon that plays a crucial role in various cognitive functions, including learning and memory. One of the key processes underlying the relationship between sleep and cognitive functions is synaptic plasticity, which refers to the ability of synapses to undergo structural and functional changes in response to experience. Synaptic plasticity is a fundamental mechanism in learning and memory, and its modulation during sleep is a subject of intense scientific investigation.

At the molecular level, several key players contribute to the intricate dance between sleep and synaptic plasticity. One critical factor is the regulation of neurotransmitters, which are chemical messengers that facilitate communication between neurons. During wakefulness, neurotransmitters such as glutamate are released, promoting synaptic strength and connectivity. However, as an organism transitions from wakefulness to sleep, there is a shift in neurotransmitter balance. One of the major neurotransmitters involved in sleep–wake regulation is adenosine. Adenosine levels gradually build up during wakefulness, leading to an increased inhibitory effect on neurotransmission. This buildup of adenosine is a consequence of the energy-consuming processes occurring in the brain during waking hours. As an individual enters sleep, adenosine levels peak, contributing to the initiation and maintenance of sleep. The interaction between adenosine and its receptors, particularly the adenosine A1 receptor, has profound implications for synaptic plasticity. Activation of the adenosine A1 receptor inhibits the release of excitatory neurotransmitters, such as glutamate. This downregulation of excitatory transmission during sleep is thought to facilitate the consolidation of memories by preventing interference from new sensory input and promoting the reorganization of existing neural connections. Another essential player in the molecular mechanisms of sleep and synaptic plasticity is the ECS. Cannabinoid receptors are abundant in regions involved in memory and synaptic plasticity. The ECS is known to be modulated during sleep, and its activation has been linked to the promotion of SWS, a deep and restorative stage of the sleep cycle [123,158,159,160].

Beyond adenosine and the ECS, other molecular players, such as growth factors and various signaling pathways, contribute to the dynamic regulation of synaptic strength and connectivity. One example is BDNF, a protein crucial for neuronal survival and growth. BDNF is involved in the processes of LTP and LTD, which are forms of synaptic plasticity associated with learning and memory. During sleep, BDNF levels have been shown to increase, supporting the consolidation of memories acquired during wakefulness [94,153,161,162,163].

## 8. Therapeutic Implications and Future Directions

The interaction between inflammatory processes, glial cells, and the ECS presents a multifaceted landscape with potential implications for sleep regulation and the manifestation of sleep disorders. Sleep disorders often coincide with alterations in glial function, specifically involving astrocytic and microglial dysregulation. Addressing these glial abnormalities at the molecular level presents a promising avenue for therapeutic intervention. 

Dysregulation in astrocytic functions, including disrupted glial transmission, could contribute to the pathophysiology of sleep disorders, particularly insomnia. Strategies aimed at enhancing astrocytic clearance mechanisms and preserving BBB integrity may mitigate neuroinflammation, fostering an environment conducive to improved sleep patterns. Also, activated microglia release pro-inflammatory cytokines, perpetuating a hyperarousal state observed in insomnia. Targeting microglial activation pathways, perhaps through the inhibition of specific signaling cascades, holds promise for attenuating the release of these cytokines, thereby promoting a less inflammatory environment conducive to better sleep.

The ECS plays a crucial role in regulating neuroinflammation and synaptic transmission. Activation of CB1R influences the release of various neurotransmitters involved in the SWC. In particular, CB1R activation on presynaptic terminals inhibits the release of excitatory neurotransmitters, such as glutamate, thereby modulating the delicate balance between arousal and sleep-promoting signals. Conversely, CB1R are abundantly expressed in GABAergic neurons, which play a pivotal role in the modulation of sleep architecture. CB1R activation on GABAergic neurons inhibits neurotransmitter release, contributing to the promotion of SWS and the suppression of REM sleep. This intricate modulation is crucial for maintaining a balanced sleep profile. The endocannabinoid tone, governed by the dynamic interplay between endocannabinoid levels and CB1R activation, is implicated in sleep regulation. Fluctuations in endocannabinoid levels, influenced by factors like stress and circadian rhythms, contribute to the modulation of CB1R activity, influencing the transition between wakefulness and sleep. CB1R activation is also linked to sleep homeostasis, the process that balances the need for sleep based on prior wakefulness. The ECS, through CB1R, interacts with sleep-promoting pathways, impacting sleep intensity and duration [131,164,165,166,167]. 

Understanding the role of CB1R activation in sleep modulation has significant implications for sleep disorder therapeutics. Dysregulation of the ECS, including alterations in CB1R signaling, has been associated with insomnia, hypersomnia, and circadian rhythm disorders. Targeting CB1R presents a potential avenue for novel interventions in sleep disorders. Despite this, challenges exist, such as the psychotropic effects associated with CB1R activation. Selective modulation of CB1R without unwanted side effects remains a goal. Future research should focus on unraveling the specific contributions of CB1R in different sleep disorders and exploring CB1R-targeted therapeutics with enhanced precision [168,169].

While historically associated with the immune system, CB2R are also expressed in the CNS, including brain regions involved in sleep regulation. The presence of CB2R on microglia, astrocytes, and neurons suggests a multifaceted role in neuroimmune interactions and sleep-related processes. Microglial activation is associated with sleep disorders, and CB2R activation has been shown to dampen microglial reactivity, potentially mitigating the neuroinflammatory component of sleep disturbances. CB2R activation exerts anti-inflammatory effects by inhibiting the release of proinflammatory cytokines from microglia. Chronic inflammation is linked to various sleep disorders, and the anti-inflammatory actions of CB2R may contribute to restoring the homeostatic balance necessary for proper sleep. Astrocytes, another glial cell type expressing CB2R, play a crucial role in maintaining neuronal health and synaptic plasticity. CB2R activation on astrocytes may modulate gliotransmission, impacting neurotransmitter availability and synaptic regulation, thereby influencing the SWC. Targeting CB2R presents potential therapeutic implications for sleep disorders characterized by neuroinflammation and dysregulated glial function. Developing CB2R-selective compounds may offer a novel avenue for interventions aiming to address the multifaceted aspects of sleep disturbances [112,114,127,128,137].

The interplay between CB2R and other components of the ECS, particularly CB1R, adds complexity to sleep regulation. Crosstalk between CB1R and CB2R may fine-tune the overall impact of endocannabinoid signaling on sleep-related neuronal pathways, suggesting a coordinated effort within the ECS. Additionally, inhibiting enzymes involved in endocannabinoid degradation, such as FAAH and MAGL, can prolong the actions of endocannabinoids, potentially influencing glial responses to inflammatory stimuli [170]. Traditional pharmacological treatments for inflammation and sleep disorders often involve nonsteroidal anti-inflammatory drugs (NSAIDs), corticosteroids, and sedative-hypnotics. These drugs act through diverse mechanisms, such as inhibiting inflammatory pathways or enhancing neurotransmitter activity. However, they may have side effects, and long-term use can lead to tolerance or dependency. ECS modulation primarily influences the immune response and glial function. Cannabinoids act on cannabinoid receptors, modulating cytokine release and inflammatory signaling. Traditional treatments, on the other hand, often target specific pathways. NSAIDs inhibit prostaglandin synthesis, corticosteroids suppress immune responses, and sedative-hypnotics enhance GABAergic neurotransmission. Both ECS modulation and traditional treatments aim to attenuate inflammation. While NSAIDs directly inhibit inflammatory mediators, cannabinoids exert broader immunomodulatory effects. ECS modulation may offer a more nuanced and targeted approach to inflammation, potentially mitigating side effects associated with non-specific inhibition of inflammatory pathways [171,172,173,174].

Furthermore, the ECS plays a crucial role in regulating the SWC. CB1R activation influences neurotransmitter release, impacting sleep architecture. Traditional sedative-hypnotics primarily target GABAergic pathways. ECS modulation may offer a more integrated and adaptable approach to sleep regulation, considering its influence on both inflammation and neural circuits governing sleep [131,175,176].

Traditional pharmacological treatments often carry side effects, such as gastrointestinal issues with NSAIDs or dependency with sedative-hypnotics. ECS modulation, particularly with cannabinoids, may have a more favorable side effect profile, although concerns about psychotropic effects and long-term consequences require careful consideration.

## 9. Conclusions

Clinical evidence supporting ECS modulation for inflammation and sleep disorders is growing. Studies on cannabinoids, especially CBD, show promising results in reducing inflammation and improving sleep quality [177,178]. Traditional treatments have a well-established track record but may lack the multifaceted effects of ECS modulation [179].

ECS modulation faces challenges related to regulatory complexities and potential psychotropic effects. Traditional treatments, while established, may not address the underlying molecular intricacies of inflammation and sleep disorders as comprehensively as ECS modulation.

The ECS–glia axis emerges as a promising target for therapeutic interventions in sleep disorders. Harnessing the regulatory capabilities of cannabinoid receptors to modulate neuroinflammation, gliotransmission, and circadian rhythms offers a nuanced approach to addressing the multifaceted nature of sleep pathology. 

Further research into the specific contributions of cannabinoid receptors in diverse sleep disorders and the development of cannabinoid receptor-selective compounds hold the potential to revolutionize treatment strategies, providing much-needed relief to individuals grappling with sleep disturbances. The evolving legal landscape, coupled with ongoing scientific research, will likely shape the integration of cannabinoids into sleep disorder management. Striking a balance between accessibility, safety, and efficacy remains a key challenge in maximizing the therapeutic potential of the ECS in the realm of sleep medicine [180,181].

As the field advances, unraveling the complexities of ECS–glia interactions will undoubtedly pave the way for innovative and targeted therapies, enhancing our ability to manage and mitigate the impact of sleep disorders on overall health and well-being.
